# Effect of Radiotherapy Combined With Pembrolizumab on Local Tumor Control in Mucosal Melanoma Patients

**DOI:** 10.3389/fonc.2019.00835

**Published:** 2019-09-04

**Authors:** Hyun Ju Kim, Jee Suk Chang, Mi Ryung Roh, Byung Ho Oh, Kee-Yang Chung, Sang Joon Shin, Woong Sub Koom

**Affiliations:** ^1^Department of Radiation Oncology, Gachon University Gil Hospital, Incheon, South Korea; ^2^Department of Radiation Oncology, Yonsei Cancer Center, Yonsei University College of Medicine, Seoul, South Korea; ^3^Department of Dermatology, Cutaneous Biology Research Institute, Gangnam Severance Hospital, Yonsei University College of Medicine, Seoul, South Korea; ^4^Department of Dermatology, Cutaneous Biology Research Institute, Yonsei University College of Medicine, Seoul, South Korea; ^5^Division of Medical Oncology, Department of Internal Medicine, Yonsei Cancer Center, Yonsei University College of Medicine, Seoul, South Korea

**Keywords:** melanoma, immunotherapy, radiotherapy, local control, adverse events

## Abstract

**Objective:** Mucosal melanoma is an aggressive malignancy with a poor response to conventional therapies. The efficacy of radiotherapy (RT), especially combined with immune checkpoint inhibitors (ICIs), for this rare melanoma subtype remains unknown. We investigated the reciprocal effect of RT and ICI on mucosal melanoma patients.

**Materials and Methods:** We identified 23 patients with 31 tumors who were treated with RT between July 2008 and February 2017. All patients received RT for primary or metastatic gross tumor mass with a median dose of 4 Gy per fraction (range 1.8–12 Gy). Eleven patients (14 lesions) were treated with RT alone, whereas 12 (17 lesions) were administered pembrolizumab combined with RT (ICI+RT group). The local control (LC) and adverse event (AE) rates were compared between the groups. Eight patients with metastatic mucosal melanoma treated with ICI alone during the same study period were included as a comparison group.

**Results:** The median follow-up period was 17.4 (range 3.7–95.2) months. The target lesion control rate at 1-year was significantly higher in the ICI+RT group than in the RT-alone group or ICI-alone group (94.1% vs. 57.1% vs. 25%; *P* < 0.05). No abscopal effect was observed in our cohort. Treatment-related AEs were not significantly increased in the combined treatment group compared with the RT-alone group (*P* > 0.05). No grade ≥3 AEs occurred in the ICI+RT group.

**Conclusions:** Besides RT acting as an immune adjuvant, ICI might have a radiosensitizing effect and may increase LC without severe toxicity. We have initiated a phase II study to determine the effects of RT in patients with melanoma undergoing anti-PD1 (NCT04017897).

## Introduction

Mucosal melanoma is a rare malignancy, accounting for only 1–4% of all melanomas (1, 2). It originates from melanocytes in the mucosal surfaces of the nasal sinuses, oral cavity, vagina, anus, and other areas. Mucosal melanoma has a behavioral pattern that is distinct from that of cutaneous melanomas. Besides its distinct presentation, mucosal melanoma has an aggressive prognosis, given its high rates of local failure and distant metastasis (3, 4). The overall 5-year survival rate is low, ranging from 20 to 30% (5, 6). As for cutaneous melanoma, wide surgical resection is the primary treatment of choice for localized mucosal melanoma. Historically, melanoma was regarded as being resistant to conventional RT (7), although this aspect remains controversial. Concerning the treatment of mucosal melanoma, a few studies have reported improved local control (LC) with postoperative RT use (8–10). However, the role of RT remained limited before the introduction of immune checkpoint inhibitors (ICIs). Systemic chemotherapy for improving treatment outcomes in mucosal melanoma remains unknown.

In the immunotherapy era, conventional assumptions regarding RT for melanoma have been challenged. Recently, ICIs have become the major treatment modality for some cancers, most distinguishably, malignant melanoma. Ipilimumab (an anti-cytotoxic T-lymphocyte antigen-4 antibody), nivolumab, and pembrolizumab (an anti-programmed-death 1 monoclonal antibody) were approved by the United States Food and Drug Administration for advanced melanoma patients (11–13). Despite the advent of ICIs, numerous patients do not respond to ICI and develop adverse events (AEs). Preclinical and clinical studies suggest that RT induces a systemic immune response to tumors outside of the radiation target (14–16). However, data analyzing the reciprocal radiosensitizing effects of ICI on LC are scarce. Furthermore, the efficacy of ICI with RT for mucosal melanoma, a rarer and more radioresistant entity of melanoma, remains unknown. We aimed to investigate the efficacy and safety of RT combined with pembrolizumab in mucosal melanoma patients.

## Methods

### Study Population

Patients treated for mucosal melanoma between July 2008 and December 2018 were identified from our institutional database. Mucosal melanomas were tumors arising from mucous membranes, such as those in the head and neck, anorectum, female genital tract, and urinary tract. Ocular melanomas, including tumors involving the conjunctiva and uvea, were excluded. Histological confirmation of the lesion was essential for all cases. We included patients receiving RT for gross tumor lesions, either at the primary site or a metastatic lesion. Among 33 patients, those not completing RT (*n* = 5) and lost to follow-up (*n* = 5) were excluded. Ultimately, 23 patients treated for 31 lesions were included. During the study period, at our institution, we then included 8 patients treated with ICI alone for mucosal melanoma to investigate the possibility of an effect of ICI on local control.

Clinical information regarding demographic data, subsite of origin, staging, surgical resection, adjuvant treatment, and outcome was retrieved. All patients were staged according to the 7th American Joint Committee on Cancer staging system for each anatomical site, given that there is no staging system that can integrate staging of mucosal melanoma. Metastasis staging was based on cutaneous melanoma criteria. This retrospective study was reviewed and approved by the institutional review board of the Yonsei University Health System (IRB 4-2017-1187). The patient records/data were anonymized and de-identified before analysis, and the requirement for informed consent was waived.

### Treatment

At our institution, the basic treatment principle for patients without distant metastasis at presentation was primary surgical resection if the lesion was resectable, followed by adjuvant interferon therapy, chemotherapy, or adjuvant RT. Adjuvant treatment was decided upon according to the disease extent and clinician's judgment. For patients diagnosed with stage IV disease, chemotherapy with dacarbazine was primarily considered before the ICI era. ICI became the first-line treatment after its approval for metastatic melanoma patients. Pembrolizumab (Keytruda®, Merck), an anti-programmed cell death-1 (PD-1) antibody, was administered intravenously at 2 mg/kg doses every 2 or 3 weeks. Local RT was considered if a solitary gross mass or symptomatic lesion existed. The radiation dose was determined depending on the anatomic site and indication. The median dose of radiation was 40 (range 20–69) Gy, with a median fractional dose of 4 (range 1.8–12) Gy. Pembrolizumab and RT administered to any lesion was considered concurrent treatment if RT was performed within 4 weeks after initiating or ending ICI; all other approaches were defined as non-concurrent treatment.

### Follow-Up

Patients were medically evaluated before administering pembrolizumab. During RT, all patients were examined once a week. Acute toxicities were recorded and graded according to the Common Terminology Criteria for Adverse Events (v4.01). After completing the scheduled treatment, regular follow-up was conducted every 1–3 months with imaging studies. All treated lesions were measured according to the Response Evaluation Criteria in Solid Tumors (RECIST) v1.1 by computed tomography or magnetic resonance imaging, as available; complete response (CR): disappearance of all target lesions; partial response (PR): ≥30% decrease in the sum of the largest diameters of the target lesions, relative to baseline; progressive disease (PD): ≥20% increase in the sum of the largest diameters of the target lesions, relative to the sum of the smallest diameter recorded, or the appearance of one or more new lesions; or stable disease (SD): neither PR nor PD (17). For patients treated with ICI alone, the response was evaluated according to the immune RECIST criteria (18). The objective response rate (ORR) was defined as the proportion of patients achieving CR or PR.

### Statistical Analysis

To compare patient demographics, *P* values were determined using the *X*^2^ test or Fisher's exact test for categorical variables and one-way analysis of variance for continuous variables. The primary endpoints were LC rates and AEs in mucosal melanoma patients treated with RT ± pembrolizumab or ICI alone. The secondary endpoints were progression-free survival (PFS) and overall survival (OS). Each endpoint was compared between the groups (ICI+RT vs. RT alone vs. ICI alone). PFS was determined from the date of initiating RT to the corresponding event, whereas OS was calculated from the date of diagnosis to the date of death or loss to follow-up. The Kaplan–Meier method with the log-rank test was used to analyze survival outcomes. All statistical tests were two-sided, with significance defined as *P* < 0.05. All data were analyzed using IBM SPSS software version 23.0 (SPSS Inc., Chicago, IL, USA).

## Results

Overall, 31 patients were analyzed in this study. Four patients received RT for two lesions each, and two patients, for three lesions sequentially. Eleven patients with 14 lesions and 12 patients with 17 lesions were included in the RT alone and ICI+RT groups, respectively. Eight patients were included in ICI alone group. Details on patient and treatment characteristics are summarized in Tables 1, 2.

**Table 1:** Patient characteristics.

	**RT alone**	**ICI+RT**	**ICI alone**	**Total**	***p***
	**(*N* = 11)**	**(*N* = 12)**	**(*N* = 8)**	**(*N* = 31)**	
Sex					0.820
Male	5 (45.5%)	2 (16.7%)	3 (37.5%)	10 (32.3%)	
Female	6 (54.5%)	10 (83.3%)	5 (62.5%)	21 (67.7%)	
Age, year	55.0 ± 12.2	58.5 ± 15.1	59.5 ± 8.6	55.1 ± 13.2	0.438
Subtype					0.012
H&N	8 (72.7%)	5 (41.7%)	7 (87.5%)	20 (64.5%)	
Anorectum	3 (27.3%)	4 (33.3%)	1 (12.5%)	8 (25.8%)	
Genitourinary tract	0 (0.0%)	3 (25.0%)	0 (0.0%)	3 (9.7%)	
BRAF mutation					0.167
No	3 (27.3%)	6 (50.0%)	5 (62.5%)	14 (45.2%)	
Yes	1 (9.1%)	0 (0.0%)	0 (0.0%)	1 (3.2%)	
Unknown	7 (63.6%)	6 (50.0%)	3 (37.5%)	16 (51.6%)	
C-kit mutation					
No	2 (18.2%)	4 (33.3%)	3 (37.5%)	9 (29.0%)	
Unknown	9 (81.8%)	8 (66.7%)	5 (62.5%)	22 (71.0%)	
LDH (IU/L)	190.4 ± 38.5	422.3 ± 749.2	164.5 ± 13.4	299.6 ± 521.9	0.547
Resection					1.000
No	5 (45.5%)	5 (41.7%)	8 (100%)	10 (43.5%)	
Yes	6 (54.5%)	7 (58.3%)	0 (0.0%)	13 (56.5%)	
Adjuvant IFN					1.000
No	10 (90.9%)	11 (91.7%)		21 (91.3%)	
Yes	1 (9.1%)	1 (8.3%)		2 (8.7%)	
No. of treated lesion	14 (35.9%)	17 (43.6%)	8 (20.5%)	39 (100%)	
RT site^*^					1.000
Primary	5 (35.7%)	7 (41.2%)		12 (38.7%)	
Metastasis	9 (64.3%)	10 (58.8%)		19 (61.3%)	
Total dose, Gy (range)^*^	45 (20–69)	36 (20–60)		40 (20–69)	0.034
Fractional dose, Gy (range)^*^	3 (2–12)	5 (1.8–8)		4 (1.8–12)	0.315
EQD2, Gy (range)^*^	48 (24–88)	44 (31.3–62)		46 (24–88)	0.051

*LDH, lactate dehydrogenase; RT, radiotherapy; IFN, interferon; EQD2, equivalent dose in 2 Gy fractions*.

* *Lesion-by-lesion analysis*.

**Table 2:** Detailed characteristics of patients treated with radiotherapy.

**No**	**Primary**	**BRAF**	**c-kit**	**LDH (IU/L)**	**Initial M stage**	**Previous treatment**	**Site irradiated**	**ICI**	**RT dose**	**RT technique**	**Best response**	**Outfield response**
1	Maxilla	NR	NR	194	M1	DTIC/IFN	Primary site	No	60 Gy/30fx	IMRT	PR	PD
2	Nasal cavity	NR	NR	164	M0	DTIC/IFN	Primary site	No	69 Gy/23fx	IMRT	PR	PD
3	Nasal cavity	NR	NR	231	M0	Resection + PORT	Sacrum	No	20 Gy/5fx	IMRT	SD	PD
4	Nasopharynx	WT	WT	119	M0	No	Primary site	No	64 Gy/32fx	IMRT	PR	PD
5	Rectum	WT	NR	200	M1	No	Primary site	No	45 Gy/15fx	3D-CRT	PR	PD
6	Rectum	NR	NR	195	M1	No	Brain	No	45 Gy/15fx	3D-CRT	SD	PD
7	Buccal mucosa	NR	NR	2,013	M0	Multiple resection, DTIC	Rt. cheek mass	No	39 Gy/13fx	IMRT	PD	PD
8	Anus	WT	WT	187	M0	Resection + PORT	Rt. humerus, Lt. scapula	Pembrolizumab	37.5 Gy/5fx, 40 Gy/10fx	3D-CRT	SD, SD	PD
9	Nasal cavity	Codon 601	WT	183	M0	Resection + PORT, DTIC, Taxol/carbo	Liver, mediastinal LN, abdominal nodule	No	45 Gy/15fx, 40 Gy/10fx, 40 Gy/10fx	IMRT	SD, SD, PR	PD
10	Nasal cavity	WT	NR	204	M0	Multiple resection, DTIC	Neck node	No	66 Gy/30fx	IMRT	PR	PD
11	Anorectal	NR	NR	143	M0	Resection	Paravertebral muscle, Lt. paravertebral muscle, Rt.	No	40 Gy/10fx, 40 Gy/10fx	3D-CRT	PR, SD	PD
12	Maxilla	NR	NR	201	M0	Resection	Recurrent neck node	Pembrolizumab	50 Gy/25fx	IMRT	PR	PD
13	Buccal mucosa	NR	NR	258	M0	DTIC, Taxol/carbo	Primary site	No	50 Gy/20fx	IMRT	PR	PD
14	Vulva	NR	NR	2,799	M0	MMS, wide excision	Pelvic nodule	Pembrolizumab	45 Gy/25fx	3D-CRT	PR	PD
15	Anorectal	WT	NR	215	M0	Resection	Abdominal nodule	Pembrolizumab	20 Gy/10fx	3D-CRT	SD	SD
16	Vulva	WT	WT	212	M0	Resection, IFN, DTIC, Taxol/carbo	Spleen	Pembrolizumab	40 Gy/10fx	3D-CRT	SD	PD
17	Nasal cavity	NR	NR	145	M1	No	Primary site	Pembrolizumab	60 Gy/25fx	IMRT	PR	PD
18	Nasal cavity	WT	WT	191	M0	Resection	Primary site	Pembrolizumab	59.2 Gy/27fx	IMRT	PR	PD
19	Vagina	WT	WT	203	M0	No	Primary site	Pembrolizumab	36 Gy/6fx	IMRT	CR	SD
20	Rectum	NR	NR	229	M1	No	Primary site, pancreatic mass, lung	Pembrolizumab	30 Gy/6fx, 25 Gy/5fx, 30 Gy/6fx	IMRT	SD, PR, SD	PD
21	Urethra	WT	WT	166	M0	No	Primary site, inguinal LN	Pembrolizumab	30 Gy/6fx, 30 Gy/6fx	3D-CRT	PR, PR	SD
22	Nasal cavity	NR	NR	249	M0	Resection	Primary site	Pembrolizumab	55 Gy/25fx	IMRT	PR	SD
23	Vulva	NR	NR	271	M0	Resection	Primary site, lung	Pembrolizumab	30 Gy/6fx, 24 Gy/8fx	IMRT	SD, SD	PD

*LDH, lactate dehydrogenase; ICI, immune checkpoint-inhibitor; RT, radiotherapy; fx, fractions; WT, wild type; NR, not reported*.

### Patients Treated With Radiotherapy Alone

Eleven patients were treated with RT alone for 14 lesions, including 5 primary sites and 9 metastatic lesions. The median age was 48 (range 39–73) years. Subtype of mucosal melanoma was head and neck in 8 (72.7%) and anorectum in 3 patients (27.3%). Six (54.5%) patients previously received resection for a primary lesion. Six (54.5%) patients received one or more prior lines of systemic therapy for advanced disease. The radiation dose was a median of 3 Gy (range 2–12) per fraction and a median of 45 Gy (range 20–69) in total. BRAF mutation status was reported in 4 (36.4%) patients, and one had a BRAF mutation.

### Patients Treated With Radiotherapy Combined With Immune-Checkpoint Inhibitor

Twelve patients were administered pembrolizumab combined with RT, concurrently in 11 patients and sequentially in 1 patient for whom pembrolizumab was administered 7 months before initiating RT. The median interval between the first administration of pembrolizumab and initiating RT was 9 (range 0–214) days. The number of treated lesions were 17, including 7 primary and 10 metastatic lesions. The median age was 58 (range 32–81) years. Subtype was head and neck in 5 (41.7%), anorectum in 4 (33.3%), and genitourinary tract in 3 (25%) patients, respectively. Radiation dose was median 5 Gy (range 1.8–8) per fraction and median 36 Gy (range 20–60) in total. A BRAF mutation was reported in half of the patients in whom no BRAF mutation was detected.

### Patients Treated With Immune-Checkpoint Inhibitor Alone

Eight patients were diagnosed with stage IV disease and received ICI as the first treatment, except two patients who previously performed first-line chemotherapy. Six patients administered pembrolizumab and two patients received ipilimumab. The median age was 59 (range 48–73) years and all patients had head and neck subtype except for 1 patient who had anorectal melanoma. No BRAF mutation was detected among 5 patients whose mutation status was known.

### Infield LC and Response Rate

**Figure 1:**
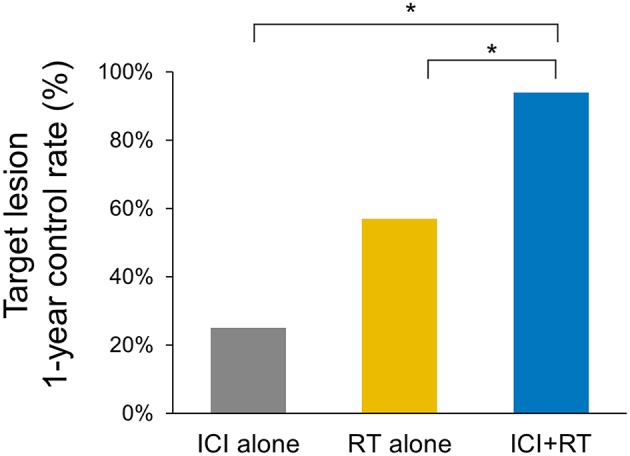
Comparison of the 1-year local control rates of the target lesion in three treatment groups (ICI+RT vs. RT alone, 94.1% and 57.1%; *p* = 0.028, ICI+RT vs. ICI alone, 94.1% vs. 25%; *p* < 0.001). ICI, immune checkpoint inhibitors; RT, radiotherapy; ICI+RT, pembrolizumab combined with RT.

**Figure 2:**
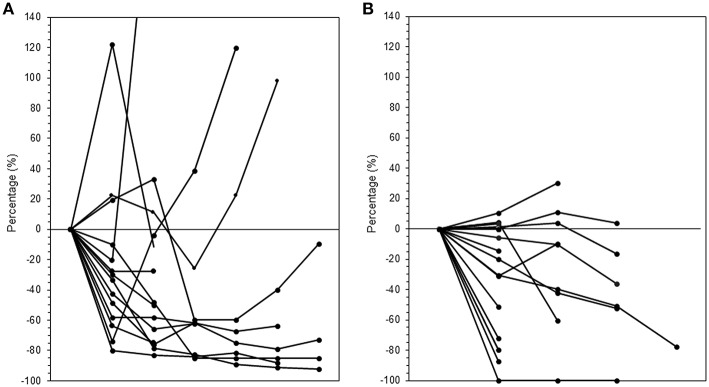
Changes in tumor volumes over time after radiation in the **(A)** RT-alone and **(B)** ICI+RT groups. ICI, immune checkpoint inhibitors; RT, radiotherapy; ICI+RT, pembrolizumab combined with RT.

**Figure 3:**
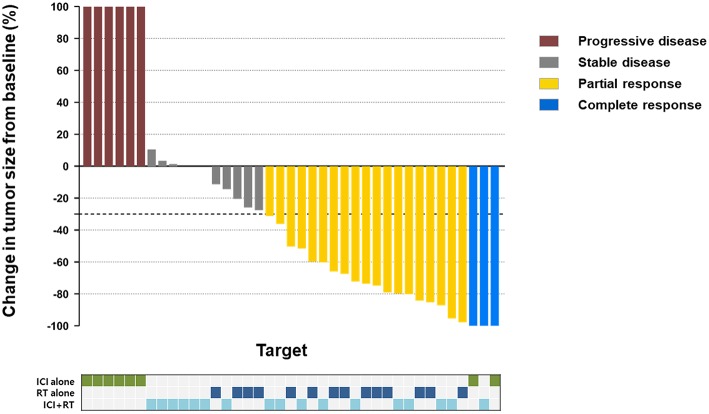
Waterfall plot of best responses of all lesions according to the treatment groups.

The median follow-up period was 17.4 (range 3.7–95.2) months. The median follow-up periods were 14 (range 3.7–95.2), 21.9 (range 9.2–56.9), and 9 (range 4–35) months for the ICI+RT, RT-alone, and ICI-alone groups, respectively. The 1-year LC rate was 94.1, 57.1, and 25% for the ICI+RT, RT-alone, and ICI-alone groups, respectively, and was significantly better in the ICI+RT combination group (*P* < 0.005; Figure 1). Of 31 lesions treated with RT, 1 showed CR and 16 showed PR, for an infield ORR of 54.8%. SD and PD were observed in 13 and 1 lesions, respectively. The infield ORRs were similar for the ICI+RT and RT-alone groups (52.9% vs. 57.1%; *P* = 0.815). However, once obtained, the response was maintained longer in the ICI+RT group than in the RT-alone group, although the follow-up period was relatively short in the ICI+RT group (Figure 2). The target lesion volume of the ICI+RT group showed decreasing tendency, whereas changes in the target lesion volume of the RT-alone group varied. Among 8 patients treated with ICI alone, 6 experienced PD and 2 had CR, with an ORR of 25%. The waterfall plot for all patients is illustrated in Figure 3.

### Treatment-Related Adverse Events

Radiation-related adverse events were observed in 12 (52.2%) of 23 patients (Table 3). Overall, the most frequently reported AE was Grade 1 or 2 fatigue. Grade 3 AEs were reported by 3 (13.0%) patients, including G3 mucositis in 2 and G3 gastrointestinal disorder in 1 patient. There was no Grade 4 or higher treatment-related AE. All AEs were transient and manageable with conservative management. Regarding AEs based on treatment sites, 8/12 patients were treated for their primary lesions, whereas 4 were treated for the metastatic mass located in the abdominopelvic area. The combination of RT and pembrolizumab did not increase AE incidence. No statistically significant differences existed in the incidence of any AEs between the two groups. Among patients treated with ICI alone, there was no grade 3 or higher AE observed.

**Table 3:** Radiation-related adverse events.

	**Adverse events**										
	**RT alone (*****N*** **=** **11)**					**ICI+RT (*****N*** **=** **12)**					***P* value**
**CTCAE category**	**Gr 1**	**Gr 2**	**Gr 3**	**Gr 4+**	**All (%)**	**Gr 1**	**Gr 2**	**Gr 3**	**Gr 4+**	**All (%)**	
General disorders (Fatigue, anorexia)	6	0	0	0	6 (54.5)	2	2	0	0	4 (33.3)	0.305
Gastrointestinal disorders	0	1	1	0	2 (18.2)	1	2	0	0	3 (25.0)	1.000^*^
Oral mucositis	0	2	2	0	4 (36.4)	1	1	0	0	2 (16.7)	0.371^*^
Xerostomia	0	3	0	0	3 (27.3)	1	1	0	0	2 (16.7)	0.640^*^
Hepatobiliary disorders	0	0	0	0	0 (0.0)	1	0	0	0	1 (8.3)	1.000^*^
Skin and subcutaneous tissue disorders	0	0	0	0	0 (0.0)	1	0	0	0	1 (8.3)	1.000^*^

*CTCAE, Common Terminology Criteria for Adverse Events*.

* *Fisher's exact test*.

### Survival Outcomes

**Figure 4:**
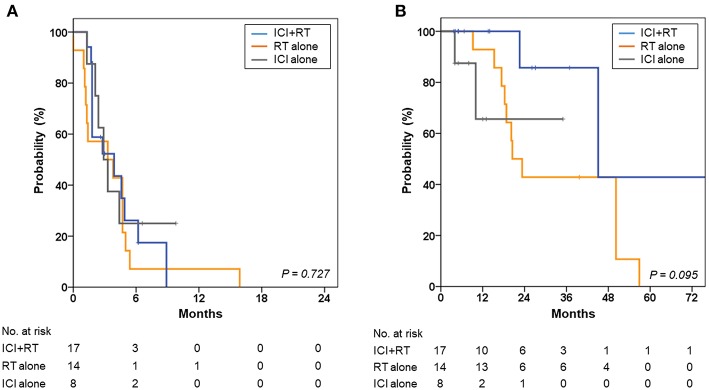
Kaplan-Meier curves for **(A)** progression-free survival and **(B)** overall survival of the ICI+RT, RT alone, and ICI alone groups. ICI, immune checkpoint inhibitors; RT, radiotherapy; ICI+RT, pembrolizumab combined with RT.

The 1- and 2-year OS rates were 90.4 and 56.0%, respectively, for all patients. The 2-year OS rates were 85.7, 42.9, and 65.6% for the ICI+RT, RT-alone, and ICI-alone groups, respectively; however, the difference was not statistically significant (*P* = 0.095; Figure 4). The 1-year PFS rates were 0, 7.1, and 25.0% for the ICI+RT, RT-alone, and ICI-alone groups, respectively (*P* = 0.727; Figure 4). Almost all patients experienced outfield tumor progression during the follow-up period. No abscopal effect was observed in our cohort. Clinical factors including gender, age, subtype, and lactate dehydrogenase were not significantly associated with OS or PFS. We could not include BRAF and c-kit mutations in the analysis as the mutational status was not reported in more than half of the patients.

## Discussion

Mucosal melanoma has biological and epidemiological features distinct from those of cutaneous melanoma. In the management of cutaneous melanoma, a remarkable evolution has recently occurred with the introduction of ICI. Response rates have improved (20–45%) in metastatic melanoma patients treated with anti-PD-1 monotherapy (12, 18, 19). However, because of its rarity, only a few studies have investigated the efficacy of ICI in mucosal melanoma (20–22). Shoushtari et al. revealed that the ORR of anti-PD-1 agents nivolumab or pembrolizumab was 23% in mucosal melanoma patients, with a median PFS of 3.9 months, which was comparable to previously published rates in cutaneous melanoma patients (20). D'Angelo et al. isolated a mucosal melanoma subtype from cutaneous melanoma and revealed that metastatic mucosal melanoma patients benefitted from ICI (22). Altogether, the current evidence implies that the response rate for ICI may not be different in mucosal melanoma, although the overall prognosis is poorer for mucosal melanoma patients.

To improve ICI efficacy, it is combined with other treatment modalities, and RT can be one of optimal candidate. Among various cancer histologies, melanomas are intrinsically radioresistant, as shown by cell culture studies (23). Interestingly, recent evidence suggests the possibility that immunity could also affect LC. Lee et al. found that RT induced upregulation of the immune response by increasing T-cell infiltration into the tumor microenvironment (TME), which induced significant tumor regression in wild-type mice, whereas tumors remained radioresistant to RT in nude mice, in which T cells were absent (24). This result suggests that the immune system is crucial for eliciting therapeutic effects of RT. Furthermore, in radioresistant tumors, namely, melanoma, whether the activation of the immune system improves the clinical efficacy of RT in terms of LC needs to be clarified.

For mucosal melanoma, wide surgical excision is the only effective approach to control macroscopic disease spread, and RT may be used selectively to control the microscopic disease. Several series have reported a suboptimal LC rate with RT alone in unresectable mucosal melanoma cases. The Northern Japan Radiation Therapy Oncology Group reported about 31 patients treated with definitive RT using a 50-Gy median dose for unresectable head and neck mucosal melanoma. The CR and PR rates were 29 and 58%, respectively, with a tumor control rate of 61% (25). Another study showed a 3-year actuarial LC rate of 49% using a radiation dose of 50–55 Gy in 15–16 fractions (26). In a randomized study comparing the effectiveness of two different RT schedules in treating soft-tissue and nodal metastasis, the ORR was 59% and CR rate was 24% (27). In our study, the ORR for RT was 54.8% for all gross lesions, which is similar to previous results. The response rate of RT is high by itself, given that the response rate in the previously reported melanoma trials of PD-1 blockade with nivolumab or pembrolizumab was 23–31% (22, 28, 29). RT may be a good treatment modality for LC when the tumor is unresectable, as is common in clinical practice.

Various preclinical studies have demonstrated that RT interacts with the immune system (14, 30, 31). This interaction can stimulate and suppress the immune system, depending on the radiation volume, dose fraction size, and tumor environment. RT causes the death of tumor cells and improves the ability of the immune system to recognize tumors by releasing tumor-associated antigens and immunostimulatory compounds within the tumor (14, 16). Sub-ablative doses of RT could increase dendritic cell activation and T-cell priming, and low-dose radiation can increase tumor-infiltrating lymphocytes by breaking down the stroma and inducing macrophage differentiation. Thus, the invasion of primed CD8+ T cells and other effector cells into the TME is promoted, providing a localized antitumor immune response. Furthermore, as previously reported in 2012 (32), immune effector cells locally primed by RT recognize and destroy tumors in non-irradiated sites that share the same antigens they are primed against—an abscopal effect.

Considering these effects of RT on immune system function, both locally and systemically, studies have focused on the clinical implication of RT combined with systemic ICI, which reverses the immunosuppressive effect of tumors, thus facilitating anti-tumor immunity together with RT. A few studies have reported the outcomes of combination treatment for cutaneous melanoma. Hiniker et al. reported that 11 of 22 patients treated with ICI+RT experienced better than SD, suggesting that a subset of patients benefit from combination therapy (33). Twyman-Saint Victor et al. reported that 18% of 22 patients had PR in a non-irradiated site when treated with hypofractionated RT followed by ipilimumab (34). In our study, despite subablative or palliative doses, the 1-year LC rate was 94.1% for the ICI+RT group, which was significantly higher than that for the RT-alone group, implying a radiosensitizing effect of ICI. Considering that the 1-year LC rates were 25% in patients treated with ICI alone, we can suggest that this may not be due to the effect of ICI alone, further implying a synergistic effect of RT combined with ICI. However, the number of patients included in this analysis was very small; thus, future clinical trials are needed. Unfortunately, we could not assess whether RT had a synergistic effect on systemic outcomes, given that our study cohort included heterogeneous clinical situations at RT. In our study, no abscopal effect was observed, and outfield progression was not improved by combination with ICI. This result could be because most lesions were treated with conventional fractions (67.7%), by which the stimulator of the interferon genes pathway is not activated and thus interferon levels are not increased (35). Radiation-induced lymphopenia, which is more frequently observed in long-course than in short-course RT, might also be associated with this outcome. Many studies are currently ongoing to determine the optimal RT dose and site to induce a more abscopal effect.

Until now, only one study has reported the result of combined RT and anti-PD-1 therapy in seven mucosal melanoma patients (36). Regarding response rates, four of seven patients (57.1%) achieved CR or PR. In our study, three among seven lesions of the RT+ICI group and 11 among all 21 lesions achieved better than PR. This result is similar to or slightly higher than that of previous reports; however, the strength of our study is that it is the only study comparing the efficacy of combination therapy to that of RT alone.

In our study, 3 (13%) patients experienced grade 3 AEs, and no AE > Grade 4 was observed. Adding pembrolizumab to RT did not increase the rate of AEs in our patients. This result is similar to recent evidence from a prospective trial investigating the safety of multisite stereotactic body radiotherapy (SBRT) followed by pembrolizumab in metastatic solid tumors (37). The trial revealed that 10% of 62 patients experienced treatment-related severe toxicity (≥Grade 2), with a median follow-up of 5.5 months. We can assume that combined ICI+RT might not synergistically increase treatment-related toxicities, given that these results are similar to those of SBRT or pembrolizumab monotherapy. However, more results from a larger number of patients with long-term follow-up are necessary to ensure the safety of combined treatment.

Our study has limitations besides its retrospective design. First, the number of patients included was small. Because of its rarity, a limited number of studies on mucosal melanoma patients have been conducted to date, and large randomized clinical trials are difficult to organize. Second, patients treated with ICI+RT were recently treated compared to those treated with RT alone. However, this approach might include little bias given that the treatment of mucosal melanoma and the RT technique have not changed that much during this period.

In conclusion, we investigated the effect of RT combined with ICI on LC in mucosal melanoma patients, especially comparing combined treatment with RT alone. RT might be helpful in improving the LC of mucosal melanoma, either in the primary or metastatic setting. We believe that ICI might have a radiosensitizing effect and may increase LC without causing severe toxicity. Further clinical trials are warranted to refine the role of RT when combined with ICI in mucosal melanoma patients. We have initiated a phase II study to determine the effects of RT in Asian patients with melanoma who have more mucosal type than their Western counterparts undergoing anti-PD1 (NCT04017897).

## Data Availability

The datasets generated for this study are available on request to the corresponding author.

## Author Contributions

All authors listed have made a substantial, direct and intellectual contribution to the work, and approved it for publication.

### Conflict of Interest Statement

The authors declare that the research was conducted in the absence of any commercial or financial relationships that could be construed as a potential conflict of interest.
